# Effects of nitrogen deposition and litter layer management on soil CO_2_, N_2_O, and CH_4_ emissions in a subtropical pine forestland

**DOI:** 10.1038/s41598-020-65952-8

**Published:** 2020-06-02

**Authors:** Jianling Fan, Ruyi Luo, Brian G. McConkey, Noura Ziadi

**Affiliations:** 1 Jiangsu Key Laboratory of Atmospheric Environment Monitoring and Pollution Control, Jiangsu Collaborative Innovation Center of Atmospheric Environment and Equipment Technology, School of Environmental Science and Engineering, Nanjing University of Information Science and Technology, 219 Ningliu Road, Nanjing, 210044 China; 2 State Key Laboratory of Soil and Sustainable Agriculture, Institute of Soil Science, Chinese Academy of Sciences, Nanjing, 210008 China; 3 Swift Current Research and Development Centre, Agriculture and Agri-Food Canada, Swift Current, SK S9H 3X2 Canada; 4 Quebec Research and Development Centre, Agriculture and Agri-Food Canada, Quebec City, QC G1V 2J3 Canada

**Keywords:** Biogeochemistry, Environmental sciences

## Abstract

Forestland soils play vital role in regulating global soil greenhouse gas (GHG) budgets, but the interactive effect of the litter layer management and simulated nitrogen (N) deposition on these GHG flux has not been elucidated clearly in subtropical forestland. A field trial was conducted to study these effects by using litter removal method under 0 and 40 kg N ha^−1^ yr^−1^ addition in a subtropical forestland in Yingtan, Jiangxi Province, China. Soil CO_2_ emission was increased by N addition (18–24%) but decreased by litter removal (24–32%). Litter removal significantly (*P* < 0.05) decreased cumulative N_2_O emission by 21% in treatments without N addition but only by 10% in treatments with 40 kg N ha^−1^ yr^−1^ addition. Moreover, litter-induced N_2_O emission under elevated N deposition (0.094 kg N_2_O-N ha^−1^) was almost the same as without N addition (0.088 kg N_2_O-N ha^−1^). Diffusion of atmospheric CH_4_ into soil was facilitated by litter removal, which increased CH_4_ uptake by 55%. Given that the increasing trend of atmospheric N deposition in future, which would reduce litterfall in subtropical N-rich forest, the effect of surface litter layer change on soil GHG emissions should be considered in assessing forest GHG budgets and future climate scenario modeling.

## Introduction

Anthropogenic activities have greatly affected greenhouse gas (GHG) emissions from the terrestrial biosphere. During the last decade, atmospheric concentrations of CO_2_, N_2_O, and CH_4_ have increased at rates of 1.9 ppm yr^−1^, 0.8 ppb yr^−1^, and 4.8 ppb yr^−1^, respectively^1^. Forestland, which covers 31% of land area and contains 365 Gt of carbon (C) in soils and litter layer^2^, plays a vital role in regulating soil C and N dynamics and global GHG budgets since they mostly act as CO_2_ and N_2_O sources and CH_4_ sinks^1^.

Atmospheric nitrogen (N) deposition has increased dramatically since last century, mainly due to anthropogenic activities such as fossil fuel combustion and ammonia volatilization caused by N fertilizer application, and it is considered that this increasing trend will continue in the next few decades^3^. Increased N availability will significantly influence soil C and N dynamics, thus altering the exchange of GHGs between the biosphere and the atmosphere^4–6^. Simulated N deposition mostly resulted in decreased CO_2_ emission by inhibiting soil autotrophic and/or heterotrophic respiration and the decomposition of soil organic C (SOC)^5,7^. Nitrate (NO_3_^−^) could increase soil redox potential and thus decrease CH_4_ production, while NH_4_^+^ may inhibit CH_4_ oxidation by methanotrophic bacteria to CO_2_^8^. Increased soil N availability from N deposition greatly increased soil N_2_O emissions. Liu and Greaver^4^ found that N addition (10–562 kg N ha ^−1^ yr^−1^) significantly increased N_2_O emission by 216% on average across different ecosystems by conducting a meta-analysis. A positive linear relationship between N rates and N_2_O emission from the subtropical forest soils was primarily due to the promotion of soil denitrification rates caused by increased N availability^9,10^. However, contrary effects or lacks of response of CO_2_, CH_4_, or N_2_O emission to elevated N deposition have also been reported^8,11^.

In forest ecosystems, the litter layer, which contributes the largest C and nutrients input to soils, plays a vital role in regulating soil C and N dynamics and GHG emission. In temperate forests, litter layer decomposition contributes about 5% to 45% of total soil CO_2_ emissions^12,13^. Litter layer removal may decrease soil fungi: bacteria ratio and then affect soil CO_2_ emissions, since litter layer decomposition is governed by fungi that can decompose cellulose and lignin^14^. Furthermore, well-aerated forest soils are considered as important CH_4_ sinks because of the CH_4_ consumption by methanotrophic bacteria^15^. Litter layer does not emit or uptake CH_4_ by itself^12^, but may affect soil CH_4_ flux by controlling its diffusion between soil and atmosphere^16^.

The largest natural source of N_2_O is from soils under natural vegetation, which accounted for 6.6 Tg N_2_O-N yr^−1^ of global terrestrial N_2_O emissions^1^. However, the effect of litter layer management on soil N_2_O flux is not clear yet. It has been reported that litter layer removal either significantly reduced soil N_2_O emission^17–19^ or had no impacts on soil N_2_O emission in subtropical and tropical forests^20^. Firstly, litter could provide organic C and N as substrate for nitrifiers and denitrifiers, but may also simulate microbial growth and activity, thus promoting N_2_O production^21^. It has been reported that removals of litter layer reduced soil N_2_O emissions by between 6% and 34% in forest ecosystems^17,18^. Secondly, litter layer may act as a barrier, which could enhance the soil anaerobic environment and then promote soil N_2_O production. Eickenscheidt and Brumme^22^ found that low soil gas diffusivity induced by litter layer, along with high N turnover rate, promoted high N_2_O emission from acid beech forest soils, which explained 77% of the variation in N_2_O fluxes. Therefore, the effect of litter layer on soil N_2_O emission is controlled by the counterbalance between the promotion and inhibition effects mentioned above. However, to our knowledge, the distinct effect of the litter layer on soil GHG fluxes in forestland under elevated N deposition remains unclear. Therefore, precise quantification of the effect of litter layer on GHG flux with different N additions will help to understand how litter layer and N deposition influence soil processes and help to improve the biogeochemical models for GHG budget assessment.

Therefore, the objectives of this study were: (i) to quantify the effect of simulated N deposition, litter removal, and their interaction on soil CO_2_, N_2_O, and CH_4_ emission; and (ii) to understand the key factors regulating soil GHG emissions in subtropical forestland.

## Results

### **Climate and soil environmental variables**

**Figure 1:**
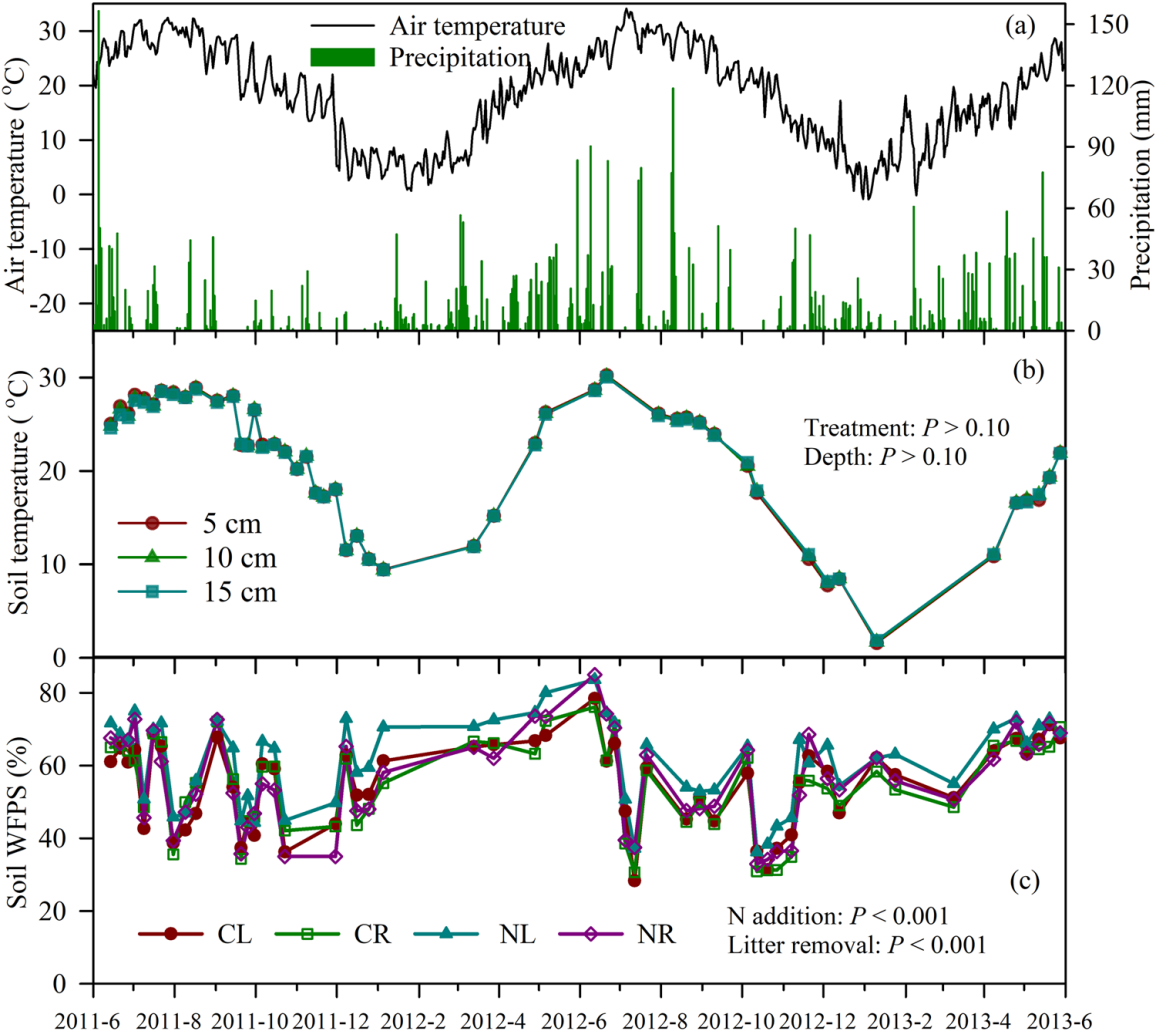
Temporal variations in daily air temperature and precipitation (**a**), soil temperature at different soil depth (**b**), and soil water-filled pore space (WFPS) at 5 cm depth (**c**) for different treatments over a 2-year period from 2011 to 2013. CL, no N addition with litter retention; CR, no N addition with removed litter layer; NL, 40 kg N ha^−1^ yr^−1^ addition with litter retention; NR, 40 kg N ha^−1^ yr^−1^ addition and removed litter layer.

Mean air temperatures in the June 2011–May 2012 (18.2 °C) and June 2012-May 2013 (18.2 °C) periods were higher than the long-term MAT (17.8 °C), while daily mean air temperatures ranged from −0.88 °C on 4 January 2013 to 34.10 °C on 6 July 2012 during the 2-yr study period (Fig. 1a). Total precipitation during the 2011–2012 and 2012–2013 periods were 2116 and 2409 mm, respectively (Fig. 1a), which mainly fell during March to September, accounting for between 69% and 81% of the annual amount.

Annual and seasonal dynamics of soil temperature at 5 cm, 10 cm, and 15 cm depths followed daily air temperature (linear relationship, *r* = 0.894–0.898, *P* < 0.001), which was not affected by different treatments (*P* > 0.05, Fig. 1b). Soil WFPS varied from 21.7% to 93.4% with a mean of 59.3% in the March to September period (rainy season), which was significantly higher (*P* < 0.001) than that from October to February (from 26.6% to 81.4% with a mean of 51.5%, Fig. 1c). Soil WFPS dynamic was primarily governed by accumulated precipitation between the two gas measurements intervals for all treatments (*r* = 0.53–0.55, *P* < 0.001). Soil WFPS was significantly influenced by N addition and litter removal (*P* < 0.001) that N addition treatments (NL and NR) showed 7% higher WFPS than without N treatments (CL and CR) and litter retention treatments (CL and NL) showed 5% higher WFPS than litter removal treatments (CR and NR).

**Figure 2:**
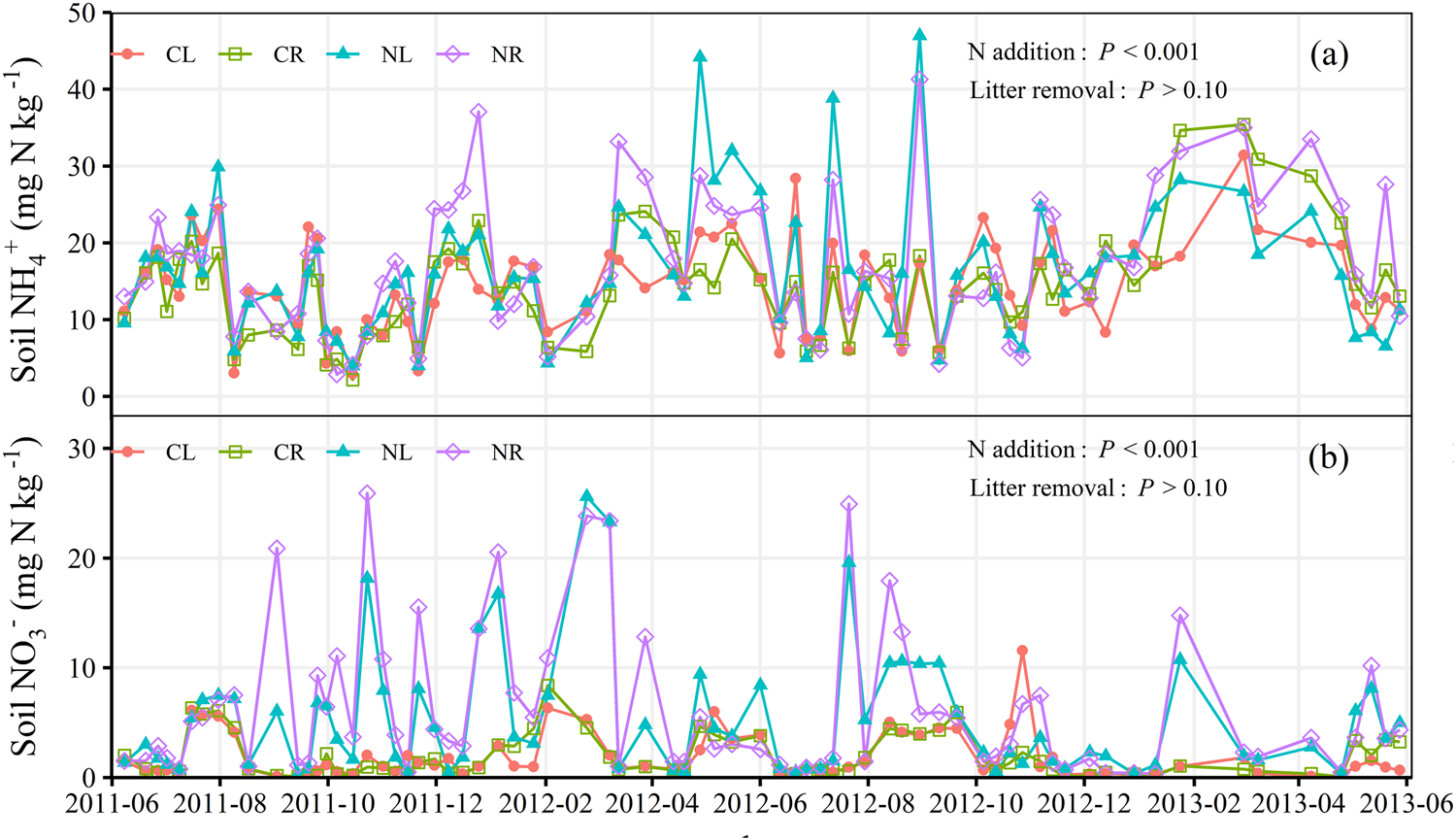
Temporal pattern of soil NH_4_^+^ (**a**) and NO_3_^−^ (**b**) concentrations (0–20 cm) over a two-year period from 2011 to 2013. CL, no N addition with litter retention; CR, no N addition with removed litter layer; NL, 40 kg N ha^−1^ yr^−1^ addition with litter retention; NR, 40 kg N ha^−1^ yr^−1^ addition and removed litter layer.

Soil NH_4_^+^-N concentrations were 16.2 and 17.3 mg N kg^−1^ on average in the NL and NR treatments, respectively and were notably higher than those in the CL and CR treatments (14.5 and 14.4 mg N kg^−1^ on average, respectively) (Fig. 2a). Soil NO_3_^−^ concentrations in the NL and NR treatments (5.0 and 5.9 mg N kg^−1^ on average, respectively) were significantly higher than those in the CL and CR treatments (1.8 and 1.9 mg N kg^−1^ on average, respectively) (Fig. 2b). However, no significant effect of litter removal on soil NH_4_^+^ and NO_3_^−^ concentration was observed in this study (*P* > 0.05).

### Soil GHG fluxes

**Figure 3:**
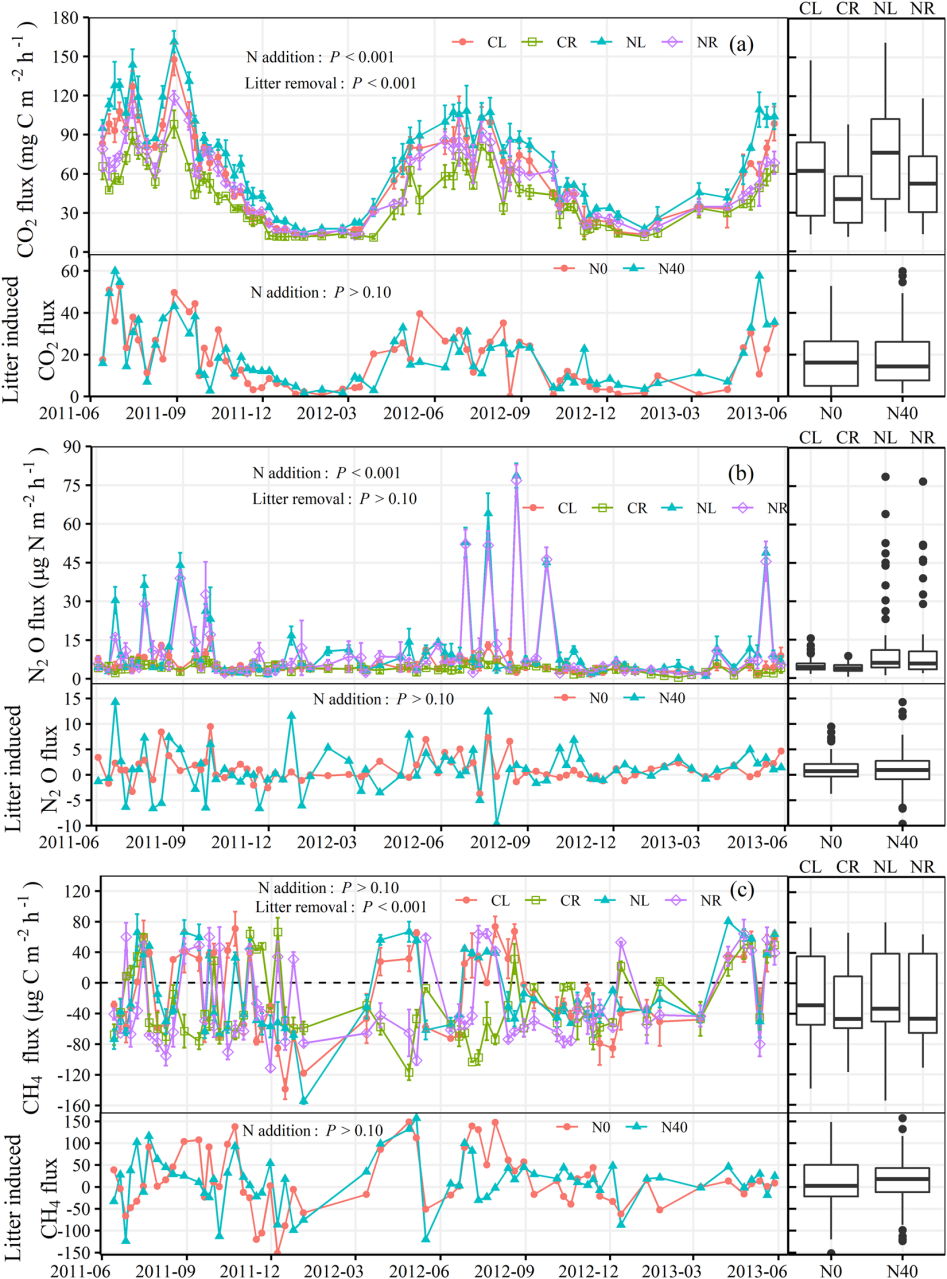
Temporal pattern of soil CO_2_, N_2_O, and CH_4_ fluxes from four treatments and litter induced CO_2_, N_2_O, and CH_4_ fluxes (*F*_CL_ – *F*_CR_ and *F*_NL_ – *F*_NR_) over a 2-year period from 2011 to 2013. Vertical bars denote the standard error (*n* = 3).

Similar seasonal and annual soil CO_2_ flux dynamics were observed among different treatments (Fig. 3a), which followed the soil temperature dynamic that decreasing from July to February (Fig. 1b; Table 1). The averaged soil CO_2_ fluxes were significantly influenced by both N addition and litter removal (*P* < 0.001), where N addition treatments (NL and NR) showed 22% higher mean soil CO_2_ fluxes than without N treatments (CL and CR) and litter retention treatments (CL and NL) showed 38% higher mean soil CO_2_ fluxes than litter removal treatments (CR and NR). Litter-induced CO_2_ flux ranged from 0.70 mg CO_2_-C m^−2^ h^−1^ in February to 59.84 mg CO_2_-C m^−2^ h^−1^ in July, while no remarkable effect of N level on litter-induced CO_2_ fluxes was observed during the study period (*P* > 0.05).

**Table 1:** Correlations between the natural logarithm of soil GHG flux (CO_2_, N_2_O, and CH_4_) and soil parameters for different treatments over the experimental period.

	Treatment	*T*_5cm_	*T*_10cm_	*T*_15cm_	WFPS	NO_3_^−^-N	NH_4_^+^-N
ln CO_2_	CL	0.884***	0.885***	0.879***	0.072	0.034	−0.006
	CR	0.839***	0.840***	0.839***	0.120	0.084	−0.134
	NL	0.832***	0.846***	0.846***	0.079	−0.198**	−0.069
	NR	0.843***	0.845***	0.844***	0.122	−0.228**	−0.122
ln N_2_O	CL	0.288***	0.293***	0.296***	0.064	0.018	−0.013
	CR	0.218**	0.218**	0.219**	−0.108	0.086	−0.150
	NL	0.151*	0.165*	0.164*	0.075	0.186*	−0.148*
	NR	0.266**	0.274***	0.268**	0.115	0.162*	−0.178*
ln CH_4_	CL	0.378***	0.384***	0.392***	0.050	0.092	−0.028
	CR	−0.092	−0.094	−0.099	0.105	0.056	0.057
	NL	0.198*	0.202*	0.200*	0.131	−0.074	0.113
	NR	−0.128	−0.127	−0.135	0.151*	0.030	0.114

CL, no N addition with litter retention; CR, no N addition with removed litter layer; NL, 40 kg N ha^−1^ yr^−1^ addition with litter retention; NR, 40 kg N ha^−1^ yr^−1^ addition and removed litter layer.

*T*_5cm_, *T*_5cm_, *T*_5cm_, are soil temperature at 5, 10, 15 cm depth, respectively; WFPS, soil water-filled pore space WFPS.

^*^P < 0.05, ^**^P < 0.01, ^***^P < 0.001.

Over the 2-yr measurement period, a sharp increase in soil N_2_O fluxes were observed after N addition, while N_2_O fluxes were mostly lower than 10.0 µg N_2_O-N m^−2^ h^−1^ for the rest of the study period (Fig. 3b). Averaged N_2_O fluxes were 5.10 ± 2.78 and 4.00 ± 1.57 µg N_2_O-N m^−2^ h^−1^ in CL and CR treatments, respectively, while N addition (40 kg N ha^−1^ yr^−1^) significantly increased N_2_O fluxes by 2.3–2.7 times. Compared with CL and NL, litter removal (CR and NR) decreased the averaged N_2_O flux by 21% and 8%, respectively, although this amount was not statistically significant. While no significant effect of N level on litter-induced N_2_O fluxes was obtained in the present study (1.11 and 1.02 µg N_2_O-N m^−2^ h^−1^ on average for N0 and N40, respectively), N addition showed much higher variation of litter-induced N_2_O fluxes than without N addition (CV of 415% *vs* 223%).

Soil CH_4_ fluxes ranged from −155 to 80 µg CH_4_-C m^−2^ h^−1^ over the study period, with 70% of observations showing negative values (Fig. 3c), indicating that the study forestland soil mainly acted as an atmospheric CH_4_ sink during the study period. Litter removal significantly increased soil CH_4_ uptake by two-fold (i.e., more negative) with average CH_4_ uptakes of 25.2–29.5 µg CH_4_-C m^−2^ h^−1^ in litter removal treatments (CR and NR) and 12.7–15.2 µg CH_4_-C m^−2^ h^−1^ in litter retention treatments (CL and NL). Furthermore, mean litter-induced CH_4_ flux was not significantly affected by N addition (*P* > 0.10).

The natural logarithms of CO_2_ and N_2_O fluxes were significantly (*P* < 0.05) correlated with soil temperature at 5, 10, and 15 cm in all treatments (Table 1). Significantly positive correlations between CH_4_ flux and soil temperature were observed in litter retention treatments (CL and NL), but negative correlations (although not significant) were observed in litter removal treatments (CR and NR). In contrast, there was no significant correlation between CO_2_, N_2_O, or CH_4_ fluxes and soil WFPS in all treatments.

### Cumulative GHG fluxes

**Figure 4:**
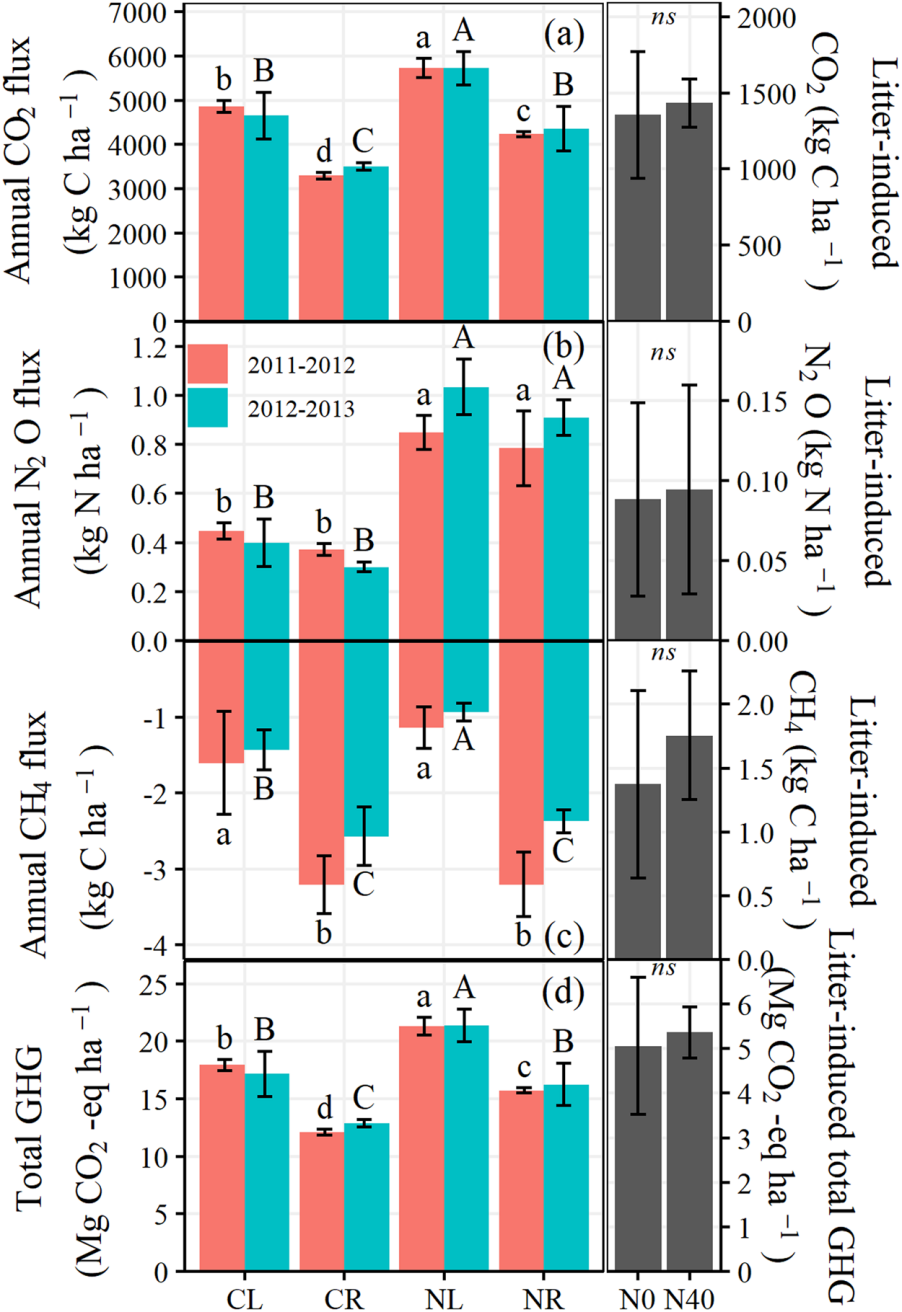
Cumulative CO_2_, N_2_O, and CH_4_ emission under different treatments over a 2-year period from 2011 to 2013. Different lower case letters and capital letters indicate significant differences among treatments at *P* < 0.05 for the 2011–2012 and 2012–2013, respectively.

Annual CO_2_ fluxes were 4858 and 4652 kg CO_2_-C ha^−1^ for CL over 2011–2012 and 2012–2013, respectively, which were 33–48% higher (*P* < 0.001) than that in CR (Fig. 4a; Table 2). The N addition treatments remarkably (*P* < 0.001) increased the cumulative CO_2_ flux to 5725–5732 kg CO_2_-C ha^−1^ for NL and to 4235–4355 kg CO_2_-C ha^−1^ for NR (18–23% and 24–29%, respectively). However, no yearly effect on annual CO_2_ flux was observed in the present study (Table 2). Litter-induced CO_2_ emissions were 1356 and 1434 kg CO_2_-C ha^−1^ in treatments with 0 and 40 kg N ha^−1^ yr^−1^ addition, respectively.

**Table 2:** The effect of N addition (N), litter removal (L), study year (Y), and their interaction on the cumulative CO_2_, N_2_O, CH_4_, and total GHG fluxes.

	CO_2_		N_2_O		CH_4_		Total GHG	
	*F*	*P*	*F*	*P*	*F*	*P*	*F*	*P*
Intercept	1532.97	<0.001	732.21	<0.001	718.95	<0.001	1538.08	<0.001
N	78.52	<0.001	264.95	<0.001	3.67	0.074	87.39	<0.001
L	174.78	<0.001	8.32	0.011	104.06	<0.001	177.76	<0.001
Y	0.08	0.780	2.29	0.151	9.21	0.008	0.14	0.711
N × L	0.14	0.717	0.01	0.925	1.51	0.238	0.15	0.704
N × L × Y	0.12	0.886	5.99	0.012	1.10	0.359	0.16	0.857

Annual cumulative N_2_O emission from June 2011 to May 2013 was significantly affected by N application (*P* < 0.01) and litter removal (*P* < 0.05) (Table 2; Fig. 4b). The lowest annual cumulative N_2_O emission was observed in the CR treatment, ranging from 0.30 to 0.37 kg N_2_O-N ha^−1^ (0.34 kg N_2_O-N ha^−1^ on average), while the highest value was obtained in the NL treatment with an average of 0.94 kg N_2_O-N ha^−1^ (0.85–1.03 kg N_2_O-N ha^−1^). Annual cumulative N_2_O emissions with N addition were 1.90–2.59 and 2.12–3.03 times higher than those without N addition for treatments with litter retention and treatments with litter layer removal, respectively. Furthermore, litter removal significantly (*P* < 0.05) decreased annual cumulative N_2_O emission by 8–25% during the study period (Fig. 4b). Litter-induced N_2_O emissions were 0.088 and 0.094 kg N_2_O-N ha^−1^ in treatments with 0 and 40 kg N ha^−1^ yr^−1^ addition, respectively.

The forest soil acted as an atmospheric CH_4_ sink from an annual perspective (Fig. 4c). Annual CH_4_ uptake in litter retention treatments (CL and NL) ranged from 0.93 to 1.60 kg CH_4_-C ha^−1^, which was 44–64% lower (*P* < 0.001) than in litter removal treatments (CR and NR) (Table 2; Fig. 4c). However, no significant (*P* > 0.05) influence of N addition on annual CH_4_ uptake was observed in the present study. Litter-induced CH_4_ emissions were 1.38 and 1.75 kg CH_4_-C ha^−1^ in treatments with 0 and 40 kg N ha^−1^ yr^−1^ addition, respectively.

Total annual GHG flux was significantly affected by both N application and litter removal (*P* < 0.001) (Table 2; Fig. 4d). Total annual GHG flux was significantly higher (*P* < 0.001) for treatments with litter retained (CL and NL, 17.2–21.4 Mg CO_2_-eq. ha^−1^ yr^−1^) than litter removal treatments (CR and NR, 12.1–16.3 Mg CO_2_-eq. ha^−1^ yr^−1^). N application (NL and NR) significantly (*P* < 0.001) increased annual GHG flux by 19–30%, compared with treatments without N addition (CL and CR). Litter-induced total GHG emissions were 5.06 and 5.36 Mg CO_2_-eq. ha^−1^ yr^−1^ in treatments with 0 and 40 kg N ha^−1^ yr^−1^ addition, respectively. Furthermore, no interaction effect of N addition and litter removal on annual CO_2_, N_2_O, CH_4_, or total GHG flux was obtained in this study (Table 2).

## Discussion

Soil respiration rates were significantly increased by N addition (between 18% and 24%) in the studied subtropical forest soil, which is in line with previously results reported based on short-term studies^23,24^. However, most of other studies reported a notable decrease of soil CO_2_ emission after long-term N addition^5,12,25,26^, mainly due to the decrease of soil microbial diversity and activity^7^, the depletion of labile C^27^, and/or reduced root biomass^28^. A 420-day incubation experiment that we conducted using the same forest soil showed that N addition significantly promoted fungal growth^29^, which may explain the increase in CO_2_ emission by N addition in the present study. However, the observed promotion of soil respiration by N addition likely represents the initial phase of the response, which may turn to decline in a long-term study. Conversely, litter removal significantly decreased CO_2_ emission by between 24% and 32%, which agrees with the reported decrease of 34% by litter removal in a meta-analysis^30^. Litter removal may reduce concentrations of dissolved organic carbon (DOC), easily mineralizable substrate for soil microbes, in both the litter layer and the mineral soil by between 22% and 31%^30^, resulting in a decline in soil CO_2_ emission.

Soil temperature was the dominant controlling factor of the seasonal CO_2_ dynamics in the present study (Table 1) and many other studies^12,31,32^. The temperature sensitivity of respiration, *Q*_10_, was in the range of 1.85–2.02 (Table 3), which fell in the lower median of the global *Q*_10_ values of 1.3 to 3.3^33^. This result indicated that the potential of temperature increase in the future may not exert a major influence on soil respiration in the subtropical *Pinus massoniana* plantation, which may be due to relative higher annual temperature in this subtropical region (annual mean air temperature of 17.8 °C) and the fact that soil respiration is more sensitive to warming in cold regions than in warm regions^34^.

**Table 3:** Relationship between CO_2_ flux and soil temperature at 5 cm depth (*T*_soil_) determined by van’t Hoff equations, and temperature sensitivity (*Q*_10_) of CO_2_ in different treatments over the experimental period.

Treatment	Equation	*Adj R*^2^	*Q*_10_
CL	*y* = 14.58 *exp*(0.067 *T*_soil_)	0.672^***^	1.98
CR	*y* = 9.38 *exp*(0.070 *T*_soil_)	0.661^***^	2.02
NL	*y* = 19.57 *exp*(0.061 *T*_soil_)	0.616^***^	1.85
NR	*y* = 13.39 *exp*(0.064 *T*_soil_)	0.655^***^	1.90
All	*y* = 14.18 *exp*(0.065 *T*_soil_)	0.546***	1.91

CL, no N addition with litter retention; CR, no N addition with removed litter layer; NL, 40 kg N ha^−1^ yr^−1^ addition with litter retention; NR, 40 kg N ha^−1^ y^r−1^ addition and removed litter layer.

^***^P < 0.001.

It has been suggested that subtropical forest soils, with an average N_2_O emission rate of 0.9–3.6 Tg yr^−1^, are an important source for the global N_2_O budget^9^, with denitrification being regarded as the main process of N_2_O production^35^, accounting for between 54% and 76% of total soil N_2_O production^36,37^. Mean annual background N_2_O emission in the CL treatment was 0.42 kg N_2_O-N ha^−1^ yr^−1^ over 2 year in the present study (Fig. 4b), which was close to the values of 0.51 kg N_2_O-N ha^−1^ yr^−1^ measured in the Notophyll vine forest of southeastern Queensland, Australia^15^ and 0.71 kg N_2_O-N ha^−1^ yr^−1^ measured in the pine plantation of Heshengqiao station in Hubei province, China^38^, but much lower than the range of 0.93–4.8 kg N_2_O-N ha^−1^ yr^−1^ reported for other subtropical forest ecosystems^9,20,37^. This low N_2_O emission may be mainly attributed to the low soil N content (0.6 g kg^−1^) of the test soil compared with other studies mentioned above (0.9–1.9 g kg^−1^), since it has been demonstrated that annual background N_2_O emission was significantly correlated with soil N and mineralized N^39^. Furthermore, much lower soil C content in the present study (0.52%) would also be responsible for the low N_2_O emission, since greater C content can enhance denitrification by stimulating the growth of denitrifying bacteria or by increasing the supply of the electron donor required by this process^40^.

The increased N deposition (40 kg N ha^−1^ yr^−1^) significantly increased N_2_O emission by 131% and 167% in treatments with and without litter, respectively (Fig. 4b). The present increase rate is lower than the results reported by Wang*, et al*.^9^, who found that 40 kg N ha^−1^ yr^−1^ addition (as NaNO_3_) increased soil N_2_O emission by 269% in a subtropical slash pine plantation in southern China. These results together suggested that soil N_2_O emissions from subtropical forestland are highly sensitive to increased nitrate deposition, which may be due to its optimal hydrothermal conditions for denitrification. Averaged soil temperature (24.2 °C) and soil moisture (60.3% WFPS) at 5 cm depth during the rainy season (Fig. 1) were within the range of the optimum denitrification condition^41^. NO_3_^−^-N input may not only supply more substrates for denitrification but also may decrease the reduction of N_2_O to N_2_ by suppressing the activity of nitrous oxide reductase^42^, which in turn increased soil N_2_O emission.

In the present study, litter removal significantly (*P* < 0.05) decreased soil N_2_O emission by 21% in treatments without N addition over 2 years (CR *vs* CL; Fig. 4b), which was in the range of 15% to 34% measured in subtropical forest^17,18^ but was lower than the range of 37% to 118% measured in temperate forest^12,43^. The contribution of litter layer to soil N_2_O emission could be mainly attributed to the mineralization of litter providing C and N substrates for nitrifiers and/or denitrifiers, thus promoting N_2_O production^21^. The lower effect of litter removal on N_2_O emission in the present study and in other subtropical forests^17,18^ compared with the temperate forest^43^ might mainly be due to the difference in litter characteristics between subtropical and temperate forests. The needle litter of subtropical forest, characterized by high polyphenol contents^44^ that would retard decomposition processes, was often less decomposable as that of temperate broad-leaved forests, especially in its early decomposition stage^45^. This finding is in line with Papen and Butterbach-Bahl^46^, who found that beech forest exhibited N_2_O emissions 4–5 times higher than that in spruce forest, indicating that forest type was an important modulator of N_2_O release from soil^47^. In contrast, litter layer removal only decreased N_2_O emission by 10% in treatments with 40 kg N ha^−1^ yr^−1^ addition, and litter-induced N_2_O emission (0.09 kg N_2_O-N ha^−1^) under elevated N deposition (NR *vs* NL) was almost the same as that without N addition (CR *vs* CL; Fig. 4b). Our results suggested that the promotion effect of N addition on N_2_O emission might be primarily derived from the enhancement of mineral soil N_2_O emission rather than from litter decomposition and corresponding N_2_O emission. The mineral soil was considered as the predominant contributor to N_2_O emission in forest ecosystems^17,20^. NO_3_^−^-N input in the present study may supply more substrates for soil denitrifiers and promote corresponding N_2_O emission. However, the insignificant effect of N addition on litter-induced N_2_O emission may be due to the fact that the test acid soil (pH = 4.64) may not be favorable for litter decomposition. Litter layer had been characterized by its low turnover rate expressed by a high mean residence time of 19 years and only 8% of forest litter layer decayed in two years during a ^15^N experiment^48^. Therefore, the effect of increased N deposition on litter layer decomposition and corresponding N_2_O emission may not be observed in a relatively short study period, such as our 2-yr study. Therefore, with the increase of atmospheric N deposition in subtropical forests, elevated N deposition may promote soil N_2_O emission by increasing its emission from mineral soils but not by stimulating litter-induced N_2_O emission.

It has been reported that N deposition may increase^26^, decrease^31^ or have no effect^25^ on soil CH_4_ flux. In our study, CH_4_ flux was not significantly affected by N addition but was remarkably influenced by litter removal, which resulted in 55% higher CH_4_ uptake in litter removal treatments. However, there are two potential explanations for the significant effect of litter removal on CH_4_ uptake. Firstly, the monoterpenes released from decomposition of pine needles^49^ may constrain the methanotrophs, then reducing the CH_4_ consumption (40–100%) by soils^50^. Secondly, litter layer may affect soil CH_4_ emission or uptake by controlling CH_4_ diffusion between soil and atmosphere^16^. Therefore, litter layer may act as a physical barrier against CH_4_ diffusion into the soil, thus reducing CH_4_ uptake in litter retention treatments. However, only net CH_4_ fluxes, rather than CH_4_ diffusion, were determined by static chamber in the present study, where further study is needed to verify this assumption.

Emission of 1 kg of N_2_O to the atmosphere is 298 times more effective than 1 kg of CO_2_, while 1 kg of CH_4_ is 34 times more effective than 1 kg of CO_2_^1^. Therefore, the GWP of the three GHGs was calculated to identify the effect of N deposition and litter removal on global warming. Our results suggested that CO_2_ was the predominant GHG in terms of GWP. In addition, significant effects of N deposition and litter removal on total GHGs were observed (Table 2; Fig. 4d), which was in line with the effect on CO_2_ emission.

Increased N deposition has been expected to stimulate C sequestration in forests, where N deposition induced forest C sinks were estimated as 0.24 to 2.0 Pg C yr^−1^ by global biogeochemical models^51^. However, Quinn Thomas*, et al*.^52^ found that tree C storage in response to N deposition was dependent on tree species, where N deposition could decrease *Pinus resinosa* growth by 9% per kg N ha^−1^ yr^−1^ but enhanced the growth of 11 tree species as high as 16–18% per kg N ha^−1^ yr^−1^. By conducting a meta-analysis, Chen*, et al*.^53^ found that N addition (50 to 150 kg N ha^−1^ yr^−1^) could decrease soil pH by 6.4%, which could directly damage root growth and inhibit tree growth, thus resulting in a 12.4% reduction of litter fall in N-rich subtropical forest. Our study site is located in south China, where, along with southwest China, has become the third-largest acid rain region in the world since 1990s^54^ and has received quite high level of N deposition since last decade^55,56^. Hence, a reduction of litter input in response to increased N deposition in subtropical pine forestland could be expected in future. Furthermore, extreme events may occur more and more frequently in future, which could also lead to either dramatic increase in litter fall input after hurricanes or severe storms^57^, or rapid loss of litter layer after wildfires^58^. Therefore, expected decrease of litter input in subtropical conifers forestland would decrease soil CO_2_ and N_2_O emission but promote CH_4_ uptake as showed in the present study. It will be essential to consider the effect of surface litter layer change on soil GHG emissions in assessing forest GHG budgets and future climate scenario modeling.

**Figure 5:**
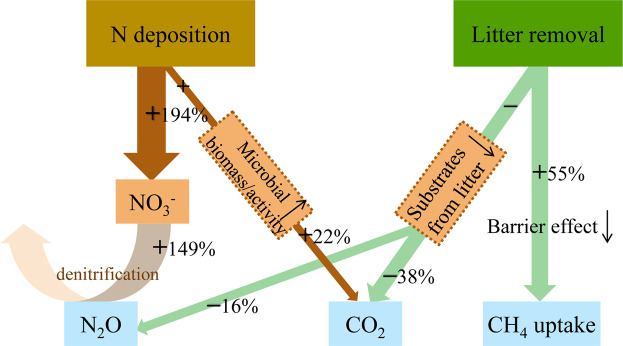
A stylized framework illustrating the main effect of N deposition and litter removal on soil CO_2_, N_2_O, and CH_4_ fluxes based on the mean values across 2-year period.

An illustration summarizing the different effects of N deposition and litter removal on soil CO_2_, N_2_O, and CH_4_ emissions is presented in Fig. 5. Simulated N deposition promoted soil N_2_O emission possibly by increasing denitrification substrates (NO_3_^−^) and promoted soil respiration by boosting microbial biomass and/or activity. Litter removal decreased the supply of C and N substrates that decomposed from litter layer, thus suppressing soil CO_2_ and N_2_O emissions. Furthermore, CH_4_ uptake was only affected by litter removal since litter layer acts as a barrier against CH_4_ diffusion. However, no interaction effect of N addition and litter removal on annual CO_2_, N_2_O, CH_4_, or total GHG flux was observed in this study. Our results indicated that N deposition and litter layer influenced soil GHG emissions via different physical or chemical processes, which should be taken into account when quantifying GHG budgets for terrestrial ecosystems.

## Methods

### **Site description and experimental design**

A field experiment was conducted at Yingtan Ecological Experimental Station of Red Soil, Chinese Academy of Sciences, Yingtan, Jiangxi Province, Southeastern China (116°55′E, 28°15′N). The area is a hilly red soil region with a typical subtropical monsoon climate, where mean annual precipitation (MAP) is 1785 mm, mean annual air temperature (MAT) is 17.8 °C. The annual accumulative temperature (>10 °C) is 5528 °C with 262 days free of frost. The study site is a 30-year-old pine (*Pinus massoniana*) plantation with an average canopy height of 5 m and a stand density of 2600 stems ha^−1^. Annual atmospheric wet N deposition is 20 kg N ha^−1^ yr^−1^ according to our field observation^56^. The soil is characterized by an acid loamy clay texture with 36% clay, 43% silt, and 21% sand, and classified as Ferric Acrisols based on the USDA soil taxonomy. Before the experiment, the soil (0–20 cm) had a pH of 4.64 and a CEC of 84.22 mmol kg^−1^ and contained 5.23 g kg^−1^ organic C, 0.63 g kg^−1^ total N, 1.68 mg kg^−1^ NO_3_^−^-N, and 1.63 mg kg^−1^ NH_4_^+^-N.

Two N levels of 0 and 40 kg N ha^−1^ yr^−1^ were established in the forest stand in 2011 to stimulate a future increase in atmospheric N deposition. To investigate the influence of litter layer on soil GHG emission, litter layer was removed using a method involving placing nylon nets (2 mm mesh) 50 cm above the soil surface after removing all detritus from the soil surface. In order to reduce soil disturbance, litter layer was removed carefully more than 1 month before the initiation of flux measurement. Fresh litter collected by nylon nets was removed once or twice per week during the study period. Therefore, four treatments were included in the present study: no N addition with litter retention (CL); no N addition with removed litter layer (CR); 40 kg N ha^−1^ yr^−1^ addition with litter retention (NL); and 40 kg N ha^−1^ yr^−1^ addition and removed litter layer (NR). Each treatment was replicated three times. A total of 12 individual plots (3 m × 3 m) were selected on the flat area with a randomized block design with a 3-m-wide buffer strip surrounded each block. N (as NaNO_3_) was weighed, mixed with 5 L of distilled water (equivalent to 0.56 mm precipitation), and applied to the NL and NR plots below the canopy using a sprayer. The solution was sprayed equally from March to September (rainy season), beginning in June 2011 and continuing throughout the study period. The same amount of distilled water was sprayed to CL and CR plots simultaneously.

### Measurement protocols

Soil CO_2_, N_2_O, and CH_4_ fluxes were determined using the closed-chamber method over a 2-yr period from 3 June 2011 to 28 May 2013 as reported by Fan*, et al*.^10^. Samples were taken in the morning between 09:00 and 12:00 once a week during the rainy season (March-September) and biweekly at other times. Concentrations of CO_2_, N_2_O, and CH_4_ in samples were measured with a gas chromatograph (Agilent 7890, Santa Clara, CA, USA) equipped with a thermal conductivity detector (TCD) for CO_2_, a ^63^Ni electron capture detector (ECD) for N_2_O, and a flame ionization detector (FID) for CH_4_. The standards were purchased from the National Research Center for Certified Reference Materials, Beijing, China. GHG fluxes were calculated using a linear least squares fit to the four sampling points for each plot, where they were omitted if the fitting had *R*^2^ < 0.90. Litter-induced CO_2_, N_2_O, and CH_4_ fluxes were calculated as the difference between treatments with litter layer and treatments with removed litter layer (*F*_CL_ vs. *F*_CR_ and *F*_NL_ vs. *F*_NR_). Cumulative fluxes were calculated by linear interpolation between measurement days.

Meteorological parameters, including daily air temperature and precipitation, were obtained from a nearby weather station (Milos 520, Vaisala, Finland). On every gas-sampling occasion, soil temperature (at 5, 10, and 15 cm) was determined using a digital thermometer, while soil water content was measured using a time domain reflectometry (TDR) probe at 5 cm depth (except when soil was frozen). Volumetric soil water content was converted to water-filled pore space (WFPS) according to the following equation:

$$WFPS=volumetric\,water\,content\,( \% )/total\,soil\,porosity\,(c{m}^{-3}\,c{m}^{-3})$$

where total soil porosity = 1 − soil bulk density (g cm^−3^)/2.65, with 2.65 g cm^−3^ being the assumed particle density of the soil.

Soil samples (0–20 cm) were collected weekly for the measurement of NH_4_^+^ and NO_3_^−^ concentrations.

### Data calculation and statistical analysis

Fluxes of CO_2_, N_2_O, or CH_4_ were calculated using a linear regression of GHG concentrations to four sampling time for each plot, by considering the chamber air temperature and atmospheric pressure. Cumulative CO_2_ (*E*_CO2_, kg CO_2_-C ha^−1^), N_2_O (*E*_N2O_, kg N_2_O-N ha^−1^), or CH_4_ (*E*_CH4_, kg CH_4_-C ha^−1^) fluxes were calculated according to the following equation:

$${E}_{C{O}_{2}}(or\,{E}_{{N}_{2}O}\,or\,{E}_{C{H}_{4}})=\mathop{\sum }\limits_{i=1}^{n}({F}_{i}+{F}_{i+1})/2\times ({t}_{i+1}-{t}_{i})\times 24$$

where *F* is the CO_2_ flux (mg CO_2_-C m^−2^ h^−1^), N_2_O flux (µg N_2_O-N m^−2^ h^−1^) or CH_4_ flux (µg CH_4_-C m^−2^ h^−1^), *i* is the *i*th measurement, the term (*t*_*i*+1_ − *t*_*i*_) is the days between two adjacent sampling, and *n* is the total times of sampling.

To evaluate the net global warming impact of CO_2_, N_2_O and CH_4_ together induced by N deposition and litter management, the total GHG were calculated according to Jiang*, et al*.^31^, where they were summed up after converting N_2_O and CH_4_ fluxes to CO_2_ equivalents (kg CO_2_-eq. ha^−1^ yr^−1^) using global warming potential (GWP) values of 298 and 34 for N_2_O and CH_4_, respectively, at the 100-yr time horizon^1^.

Soil temperature, soil WFPS, and GHG fluxes (CO_2_, N_2_O, CH_4_, and total GHG) data were evaluated using mixed effects model with the *lme* function in the ‘nlme’ package, where N addition, litter removal, study year, and their interaction were treated as fixed effects, while blocks and/or sampling date were considered as random effect. GHG flux data were natural logarithm transformed as needed, to normalize the distributions prior to statistical analysis. Pearson correlation analysis was used to identify significant correlations between the natural logarithms of the GHG fluxes and the measured environmental variables with the *corr.test* function in the ‘psych’ package. The van’t Hoff equation was established to calculate the temperature sensitivity (*Q*_10_ = *exp* (10*b*)) of CO_2_ fluxes to changes in soil temperature with the *nls* function in the ‘stats’ package. All statistical effects were considered significant at *P* < 0.05. Figures were prepared by ‘ggplot2’ package. All these analyses were performed with R software^59^.

## Data Availability

All data generated or analysed during this study are included in this published article.
